# The Antidepressant-Like and Analgesic Effects of Kratom Alkaloids are accompanied by Changes in Low Frequency Oscillations but not ΔFosB Accumulation

**DOI:** 10.3389/fphar.2021.696461

**Published:** 2021-08-03

**Authors:** Shoshana Buckhalter, Eric Soubeyrand, Sarah A.E. Ferrone, Duncan J. Rasmussen, Joshua D. Manduca, M. Sameer Al-Abdul-Wahid, Jude A. Frie, Jibran Y. Khokhar, Tariq A. Akhtar, Melissa L. Perreault

**Affiliations:** ^1^ Department of Molecular and Cellular Biology, University of Guelph, Guelph, ON, Canada; ^2^ Department of Biomedical Sciences, University of Guelph, Guelph, ON, Canada; ^3^ NMR Center, University of Guelph, Guelph, ON, Canada; ^4^ Collaborative Program in Neuroscience, University of Guelph, Guelph, ON, Canada

**Keywords:** kratom, neuronal oscillations, depression, analgesia, mitragynine, ΔFosB, *Mitragyna speciosa* 3

## Abstract

*Mitragyna speciosa* (“kratom”), employed as a traditional medicine to improve mood and relieve pain, has shown increased use in Europe and North America. Here, the dose-dependent effects of a purified alkaloid kratom extract on neuronal oscillatory systems function, analgesia, and antidepressant-like behaviour were evaluated and kratom-induced changes in ΔFosB expression determined. Male rats were administered a low or high dose of kratom (containing 0.5 or 1 mg/kg of mitragynine, respectively) for seven days. Acute or repeated low dose kratom suppressed ventral tegmental area (VTA) theta oscillatory power whereas acute or repeated high dose kratom increased delta power, and reduced theta power, in the nucleus accumbens (NAc), prefrontal cortex (PFC), cingulate cortex (Cg) and VTA. The repeated administration of low dose kratom additionally elevated delta power in PFC, decreased theta power in NAc and PFC, and suppressed beta and low gamma power in Cg. Suppressed high gamma power in NAc and PFC was seen selectively following repeated high dose kratom. Both doses of kratom elevated NAc-PFC, VTA-NAc, and VTA-Cg coherence. Low dose kratom had antidepressant-like properties whereas both doses produced analgesia. No kratom-induced changes in ΔFosB expression were evident. These results support a role for kratom as having both antidepressant and analgesic properties that are accompanied by specific changes in neuronal circuit function. However, the absence of drug-induced changes in ΔFosB expression suggest that the drug may circumvent this cellular signaling pathway, a pathway known for its significant role in addiction.

## Introduction

*Mitragyna speciosa*, commonly referred to as kratom, is a tree species that is native to Southeast Asia and it has been used by individuals for centuries both recreationally and medicinally to improve mood and manage acute and chronic pain (Singh et al., 2016; Ismail et al., 2019). However, the increase of kratom sales across Europe and North America have resulted in growing concerns over its safety, with several European countries and states within the United States banning the plant or instituting age restrictions in its use (Cinosi et al., 2015). Despite these restrictions, it is estimated there are several million users of kratom (Henningfield et al., 2018). Consumption of kratom leaves has been reported to have dose-dependent effects, in that lower doses have been found to induce mild stimulant-like effects and higher doses have been found to induce opioid like analgesic effects (Kruegel and Grundmann, 2018). These outcomes of ingesting the plant material have historically been attributed to only two alkaloids that are typically present within the kratom leaf material, namely mitragynine and its derivative 7-hydroxymitragynine (7-HMG), however it is well established that there are at least 40 other alkaloids that accumulate within the plant, albeit in various amounts (Prozialeck et al., 2012; Eastlack et al., 2020). Strikingly, almost nothing is known about the biological properties of these other alkaloids, or of the combined biological effects of the plant as a whole.

The alkaloid profile observed within kratom is dominated by the class of compounds known as the monoterpenoid indole alkaloids, of which mitragynine and 7-HMG are the most studied examples. Both alkaloids have been found to activate the mu-opioid receptor (MOR), however unlike many other MOR agonists, they are ß-arrestin sparing (Kruegel et al., 2016; Ismail et al., 2019). For this reason these alkaloids have been termed “atypical opioids” (Adkins et al., 2011; Kruegel et al., 2019), and as ß-arrestin signaling has been shown to mediate opioid-induced tolerance and side effects such as respiratory depression, it is believed that kratom may offer an analgesic alternative (Takayama et al., 2002; Kruegel et al., 2016; Ismail et al., 2019).

Behaviours are highly coupled to neuronal oscillations (Buzsáki and Draguhn, 2004; Buzsáki et al., 2013), rhythmic neuronal population activity that is critical to regional communication (Fries, 2005; Barardi et al., 2014). These oscillations have been linked to neuropsychiatric disorders such as addiction and depression (Fitzgerald and Watson, 2018; Theriault and Perreault, 2019; Zhu et al., 2019; Thériault et al., 2021), as well as drug responses (Reakkamnuan et al., 2017; Manduca et al., 2020), and thus may serve as useful biomarkers of disease states or establishing the therapeutic efficacy of novel drugs. Although there is less known about the role of neuronal oscillations in the context of analgesia, high frequency cortical oscillations are thought to be involved in the perception of pain (Whittington et al., 1998; Croft et al., 2002). For instance, morphine-induced frequency-specific alterations in oscillatory activity in brain regions such as the NAc and VTA are well known (Reakkamnuan et al., 2017; Ahmadi Soleimani et al., 2018), as well as in the cortex (Liu et al., 2005; Zuo et al., 2007). It is therefore intriguing that although there is some evidence for analgesic and antidepressant properties of kratom alkaloids (Matsumoto et al., 2004; Takayama, 2004; Kumarnsit et al., 2007a; Kumarnsit et al., 2007b; Sabetghadam et al., 2010; Idayu et al., 2011; Grundmann, 2017), the impact of these alkaloids on the neuronal oscillatory activity in the brain is lacking. Two studies examining the neurophysiological effects of mitragynine in rats did, however, demonstrate frequency-specific changes in cortical oscillatory power (Yusoff et al., 2016; Thériault et al., 2020) with no effects in other regions including the VTA, NAc, thalamus, amygdala, or hippocampus (Thériault et al., 2020).

These findings suggest that kratom-induced behavioural changes will be accompanied by region-specific alterations in neurophysiological circuit function. Therefore, to better understand this link, this study evaluated the dose-dependent effects of a purified alkaloid isolate from kratom on neuronal oscillatory activity in various brain regions in rats following acute and 7 days of administration. The antidepressant and analgesic effects of the extract were also determined at both time points, as well as the ability of the isolate to induce ΔFosB expression, a putative molecular switch for addiction.

## Materials and Methods

### Animals

Adult male Wistar rats (Charles River, QC) weighing approximately 300–350 g at the start of the experiment were used. Animals were housed in a temperature-controlled colony room, maintained on a 12-h reverse light/dark cycle (0700 h lights off; 1900 h lights on) with unrestricted access to food and water available ad libitum. Animals were handled daily for a minimum of 7 consecutive days prior to the beginning of the experiment. Electrophysiological and behavioural testing was always conducted during the dark phase of the day/night cycle. An extra cohort of animals were added to increase sample size for the behavioural studies. All animals underwent identical behavioural procedures. No group differences in animals that underwent the same treatments was evident between the two cohorts and so animals were pooled. All procedures were approved by the Animal Care Committee of the University of Guelph and followed the guidelines of the Canadian Council on Animal Care.

### Plant Material and Growth Conditions

*Mitragyna speciosa* (Korth.) Havil were obtained from Dad’s Greenhouse, Ohio, United States, and imported to the University of Guelph as 6–18″ saplings. Trees were maintained in growth chambers with a 16-h photoperiod (175 µmol m−^2^s−^1^; mixed cool white and incandescent bulbs) and a day/night temperature regime of 28°C/26°C, with a constant relative humidity of 80%. Plants were grown for a minimum of 4 months in a 2:1:1 (v/v) mixture of coco coir (Millennium soils Coir):perlite (Therm-o-rock East Inc.):turface (Turface Athletics) before harvesting material. The plant material was identified and authenticated by Dr. Carole Ann Lacroix and a voucher specimen (No. 102033) was deposited at the Ontario Agricultural College Herbarium in Guelph, Ontario, Canada.

### Alkaloid Extraction and Preparation

Mature leaf tissue from *Mitragyna speciosa* plants were dried at 50°C for 48 h and 100 g of this material was extracted with 2 L of an acetic acid solution (0.5 M) at 80°C for 30 min. The crude extract was filtered (0.22 µm PTFE) and then passed through a 60 ml column containing polyvinylpyrrolidone (PVPP, 110 µm particle size, Sigma-Aldrich) to remove any polyphenolic compounds. The crude Kratom extract was sequentially chromatographed over 50 ml of Diaion HP-20 resin (Supelco) equilibrated with distilled water and the reversed-phase column was then washed with 20% (v/v) methanol before elution with 100% methanol followed by methanol/ethyl acetate (50:50 v/v). The recovered alkaloid fractions were pooled and reduced to a volume of 200 ml on a Rotary Evaporator (RE-200AA) at 70°C. The isolate was then loaded onto an ion exchange resin (AmberChrom 50WX2, 200–400, Sigma Aldrich) and washed with 500 ml of acetic acid in ethanol (0.025 M), followed by 250 ml of 100% ethanol. Alkaloids were eluted with 340 ml of 2.8 M ammonium hydroxide in ethanol and then brought to final volume of 150 ml. This purified alkaloid extract was subjected to phase separation with chloroform (300 ml). The organic layer was extracted and reduced to dryness, *in vacuo*, and resuspended in 10 ml of hydrochloric acid (0.2 M). After complete resuspension of the alkaloid extract, the pH was brought to 5.0 with NaOH and adjusted to 1.0 mg/ml of mitragyine equivalents, accordingly.

### Instrumentation and Alkaloid Analysis

Ajmalicine (Sigma) and mitragynine, 7-hydroxymitragynine, paynantheine, speciogynine, mitraphylline, speciociliatine (Cayman Chemicals) were used as external standards for quantification on the basis of peak area revealed by HPLC analysis as described below. Alkaloids fractions were analyzed using an Agilent 1,260 Infinity liquid chromatography system equipped with a reversed-phase Kinetex EVO C18 100Å column (150 × 4.6 mm, 5 µm). Chromatographic separation of kratom alkaloids were achieved using a binary gradient with ammonium bicarbonate buffer (5 mM pH 9.5; A) and acetonitrile (B), starting with 70% solvent A transitioning to 70% solvent B over the course of 17 min at a flow rate of 1.5 ml/min. Alkaloids were quantified at 226 nm. The alkaloids fractions 3 (3-isoajmalicine) and 10 (corynantheidine) eluted at 8.12 and 14.67 min, respectively, and were subsequently collected. Approximately 0.3 mg of each compound were evaporated to dryness, resuspended in deuterated chloroform, and analyzed using ^1^H NMR. NMR spectra were collected on a Bruker AVANCE III 600 MHz spectrometer equipped with a 5 mm TCI cryoprobe. The sample temperature was regulated at 298 ± 1 K.

### Drugs

Rats were intraperitoneally (i.p.) injected daily with the kratom isolate at a dose of 0.5 or 1.0 mg/kg of mitragynine equivalents (low and high dose, respectively) or with a saline for a period of seven days. As kratom is normally ingested orally, we used the i.p. route since it also has an important first pass effect but is not as stressful as oral gavage which would likely alter brain wave patterns. Moreover, since the metabolites of kratom alkaloids have been shown to have biological effects, the i.p. route would ensure that they are metabolized in a manner similar to oral intake in humans.

These doses were selected based on preliminary dose response studies and were 5 and 10 times less than calculated LD50 of tested animals (a mitragynine equivalent of 5 mg/kg). At the doses employed in the present study, animals showed no adverse effects with acute or repeated administration. We have previously characterized isolated mitragynine effects (10 mg/kg i.p., a standard dose used in the literature (Foss et al., 2020; Japarin et al., 2021; Suhaimi et al., 2021)) on neuronal oscillatory activity where we showed moderate frequency-specific changes in cortical regions only (Thériault et al., 2020). Further, our preliminary behavioural findings showed no effects of the same 10 mg/kg dose of mitragynine on behavioural responses in the tail-flick test (Supplementary Figure S1). As the 10 mg/kg dose is 10–20 times higher than the doses used in the present study, we therefore chose not to include a mitragynine group as neurophysiological and behavioural effects would likely be minimal or absent.

### Electrode Implantation Surgery

Electrode implantation surgeries were performed as previously described (Foute Nelong et al., 2019). Custom electrode microarrays were built using prefabricated Delrin templates and polyimide-insulated stainless-steel wires (A-M Systems: 791600, 0.008″) that were inserted through polyimide cannula. All arrays used had an electrode impedance of less than 2 MΩ. Isoflurane was used to anesthetize the rats at 5% induction and 2.5% maintenance and body temperature maintained at 37°C using a thermostat-regulated heating pad. Animals were injected subcutaneously with 0.9% saline (3 ml) to ensure adequate hydration during surgeries, and 5 mg/ml carprofen (0.4 ml, s.c.) as well as a lidocaine/bupivacaine injection at the incision site. Electrodes were implanted bilaterally into the medial PFC (AP: +3.24 mm, ML: ± 0.6 mm, DV: −3.8 mm), Cg (AP: +1.9 mm, ML: ± 0.5 mm, DV: −2.8 mm), NAc (AP: +1.92, ML: ± 1.2 mm, DV: −6.6 mm) and the VTA (AP: −4.8 mm, ML: ± 0.7, DV: −8.5 mm). A ground/reference screw was secured in the skull behind lambda and additional anchor screws were attached to the skull.

### Local Field Potential Recordings

Animals were habituated to the recording boxes (18″ × 18″ × 18″) for 15 min/day for 2 days. Local field potential (LFP) recordings (Wireless 2100-system, Multichannel Systems) were performed in awake and freely moving animals on days 1 and 7 with a sampling frequency of 1 kHz. On each day of testing baseline LFP recordings were collected for 15 min prior to animals receiving their assigned kratom dose (0, 0.5, 1.0 mg/kg i.p.). Rats were then placed back into the boxes and recordings were collected for an additional 30 min. Routines from the Chronux software package for MATLAB (MathWorks) were used to analyze LFP spectral power and coherence between brain regions. Recordings were segmented, detrended, de-noised and low-pass filtered to remove frequencies greater than 100 Hz. Continuous multitaper spectral power for the normalized data (to total spectral power) and coherence was calculated for delta (1–4 Hz), theta (>4–12 Hz), beta (>12–30 Hz), low gamma (30–60 Hz), and high gamma (>60–100 Hz).

### Forced Swim Test

The forced swim test (FST) is a test used to evaluate behavioural despair and to determine the antidepressant properties of drugs, and was performed as previously described (Thériault et al., 2021) immediately following LFP recordings. The pre-test was carried out twenty-four hours prior to drug administration in which animals were placed in a plexiglass cylinder with water (24 ± 1°C) filled to a height of 30 cm for 15 min. Animals were then dried with a towel and put back into their home cage. Twenty-four hours later, following LFP recordings, animals were once again placed in the water-filled cylinder for a testing period of 5 min. For subsequent testing on day 7, the pre-test was not conducted. The following behavioural parameters were assessed at 5-s intervals: immobility (floating without active movements, other than those that are needed to keep nose above water), climbing (attempting to escape the cylinder with front paws breaking the surface of the water) and swimming (paddling of limbs across the surface of the water).

### Tail-Flick Test

To assess the acute and chronic analgesic properties of kratom, the tail-flick test was performed as previously described (Tu et al., 2016) at 40 min post-drug administration. This test evaluates pain responses in animals and is used to measure the effectiveness of analgesics through heat exposure to the animals’ tails. Animals were gently restrained using a towel and the middle third of the tail was placed in the groove on the automated tail-flick apparatus (Columbus Instruments, Columbus, OH). Radiant heat from a light was applied to the underside of the tail and the time it took (in seconds) for rats to withdraw their tail from the heat source was measured as their tail-flick latency. The intensity of the radiant heat was pre-set at 15 (approximately 60°C) throughout the experiment. An average of two baseline tail-flick latencies in all animals were recorded prior to drug administration. To prevent tissue damage, a cut-off time of 10°s was used.

### ΔFosB Immunohistochemistry

Following behavioural testing on the final day animals were perfused with 4% paraformaldehyde (PFA). Brains were extracted, flash frozen and stored at −80°C. Fluorescence immunohistochemistry was performed as previously described (Perreault et al., 2012) on PFA-fixed floating coronal brain sections (30 μm). Free-floating sections from the PFC, Cg, NAc and VTA were washed in TBS (60.5 mM Tris, 87.6 mM NaCl pH 7.6), then blocked (10% goat serum, 1% BSA, 0.2% Triton-X, 1X TBS), and incubated with primary ΔFosB antibody (Cell Signalling, Catalogue #14695, 1:200) in buffer (2% goat serum, 0.01% Triton-X and 1X TBS) for 60 h at 4°C. Following incubation, the brain sections were washed in TBS, blocked (5% goat serum, 0.5% BSA, 0.01% Triton-X, 1X TBS) and incubated for 2 h at room temperature with a secondary anti-rabbit Alexa Fluor 488. After three washes in TBS, brain sections were mounted on slides with Prolong Gold (Thermo Fisher Scientific). Images were acquired by fluorescence microscopy (Etaluma Lumascope) with a 20X objective lens, and cell counting was performed to quantify the mean number of ΔFosB positive cells in two sections of each brain region.

### Statistical Analysis

LFP power analysis was performed on 30 s epochs and is reported as means ± sem. taken every 5 min. For the coherence 30s epochs were analyzed at 30 min post-injection. Quantification of the data at each frequency measure and time point is reported as mean ± sem with spectral power curves presented as normalized data (to total power) with jackknife estimates of sem. For the power time courses a repeated measures ANOVA was performed for each frequency with Time as the within subject variable and Treatment as the between-subjects variable. In case of significant interactions or main effects, group comparisons at each time point were performed using a one-way ANOVA with Treatment as the between subjects variable and was followed by Tukey’s *post-hoc* test. The Games-Howell post-hoc test was used to determine group mean differences if the data did not pass Levene’s test for homogeneity of variance. Data were removed only if the signal quality was poor. No data were removed as a result of electrode misplacement. For the FST, the data are expressed as percent change from controls whereas the tail-flick data are expressed as percent change from baseline measures taken on each day (averaged between two readings). Data on each day were analyzed using a one-way ANOVA with Treatment as the between subjects factor followed by Tukey’s post-hoc test for group comparisons. Paired t-tests were used to compare means on day 1 and day 7. For the ΔFosB data, group comparisons were performed using Student’s t-test. Prior to all analyses, normality was assessed using the Shapiro-Wilk test.

## Results

**FIGURE 1:**
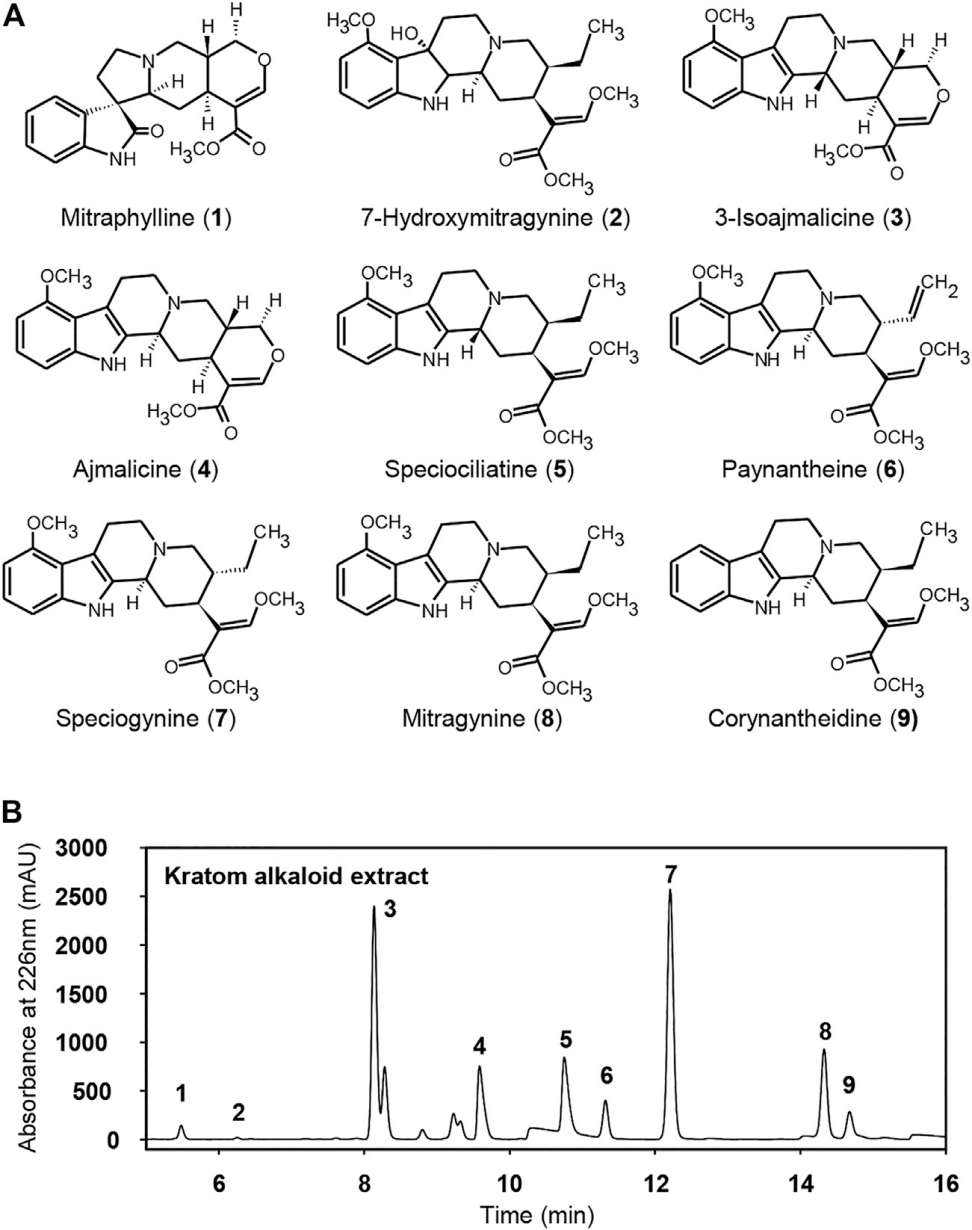
Constituent alkaloids in whole kratom extract. **(A)** Compound structures and **(B)** HPLC chromatogram of the alkaloid profile of the kratom leaves extract at 226 nm. The peaks represent respectively: 1, mitraphylline; 2, 7-hydroxymitragynine; 3, 3-isoajmalicine; 4, ajmalicine; 5, speciociliatine; 6, paynantheine; 7, speciogynine, 8, mitragynine, and 9, corynantheidine.

**FIGURE 2:**
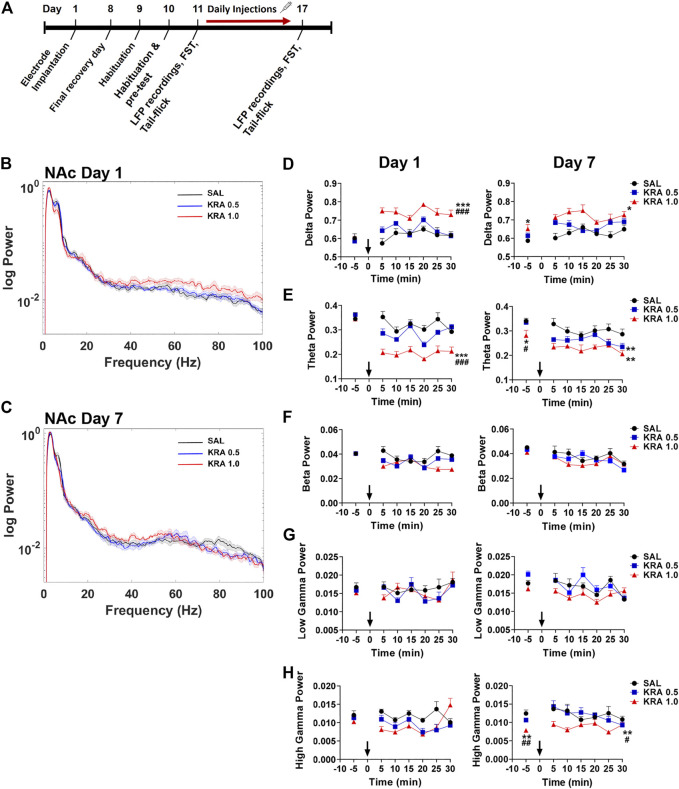
Time course of dose-dependent effects of kratom on NAc spectral power. **(A)** Experimental timeline is shown. **(B,C)** Representative power spectra showing dose-dependent (0, 0.5, 1.0 mg/kg) effects of kratom 30 min post-injection on day 1 and day 7. **(D)** High dose (1.0 mg/kg) kratom increased delta power across 30 min on both day 1 and day 7 with lasting changes observed prior to the final injection of high dose kratom. **(E)** Only high dose kratom decreased theta power on day 1 whereas both doses of kratom reduced theta power on day 7. Prior to the final injection a baseline suppression in theta power was evident in response to the high dose of kratom. **(F,G)** No effects of kratom administration on beta or low gamma power were observed on day 1 or day 7. **(H)** On day 1, no kratom-induced changes in high gamma power were observed. On day 7, high dose kratom decreased baseline high gamma power. This decrease in response to high dose kratom persisted following the day 7 injection. *N* = 7–10 rats per group, with 2 electrodes/rat. Curves are represented as means with jackknife estimates of sem depicted by the shaded areas. Bars represent mean ± sem. *p < 0.05, **p < 0.01, ***p < 0.001 compared to saline control rats. ^#^ *p* < 0.05, ^##^ *p* < 0.01, ^###^ *p* < 0.01 compared to low dose kratom treated rats.

To elucidate the dose-dependent antidepressant and analgesic effects of kratom, an alkaloid isolate was first prepared from mature kratom leaf material. The final Kratom alkaloid preparation (Figure 1) contained at least nine main annotated alkaloid species, of which seven were identified by comparison to commercially available standards: 1, mitraphylline; 2, 7-hydroxymitragynine; 4, ajmalicine; 5, speciociliatine; 6, paynantheine; 7, speciogynine, 8, mitragynine (Figure 1, Supplementary Figure S2). Alkaloids 3 and 10 eluted at 8.12 and 14.67 min, respectively, and were subsequently determined by ^1^H NMR. The ^1^H NMR spectra obtained for fractions 3 and 10 matched with those reported previously in the literature which were identified as 3-isoajmalicine and corynantheidine, respectively (Figure 1, Supplementary Figure S3,S4). This alkaloid isolate, *in toto*, was administered to rats and LFP recordings were taken to evaluate associated impacts on neural systems function, followed by assessments in the FST and tail-flick tests. These measures were evaluated following an acute injection of kratom and again following the repeated administration of the kratom isolate for 7 days. The experimental timeline is shown in Figure 2A.

### Spectral Power

#### Nucleus Accumbens

Brain rhythms, or neuronal oscillations, are highly conserved across species, are coupled to specific behavioural states, and are a key indicator of the communication status between neurons (Buzsaki and Draguhn, 2004; Buzsáki et al., 2013). The low frequency bands, delta (0.5–4 Hz), theta (>4–8 Hz), and alpha (>8–12 Hz), are slow waves and are critical in long-distance or between region communication, whereas the high frequency bands, beta (>12–30 Hz) and gamma (>30 Hz), are fast waves that play a role in short-distance or within region communication (Buzsáki and Draguhn, 2004). Power spectra depicting changes in NAc oscillatory power 30 min post-injection on day 1 and day 7 are shown (Figures 2B,C) with quantification of the spectra at 5 min time points also depicted (Figures 2D–H). There were no baseline group differences in spectral power at any frequencies (Figures 2D–H, left panels). Administration of low dose (0.5 mg/kg) kratom had no effect on delta power on day 1 or day 7. However, acute administration of high dose (1 mg/kg) kratom induced a significant increase in delta power, compared to both the low dose group (*p* < 0.001) and saline controls (*p* < 0.001), across the 30 min time period (Figure 2D, left panel) [Time: F(5,100) = 2.9, *p* = 0.016; Treatment × Time: F(10,100) = 2.4, *p* = 0.012; Treatment: F(2,20) = 29.4, *p* < 0.001]. On day 7, prior to the last kratom injection baseline delta power was elevated in those rats that received high dose kratom (*p* = 0.013) indicating potentially lingering drug effects from the day 6 injection. Following the final administration of high dose kratom this increase was maintained across the recording period (Figure 2D, right panel) [Time: F(5,150) = 4.1, *p* = 0.001; Treatment × Time: F(10,150) = 2.2, *p* = 0.020; Treatment: F(2,30) = 4.7, *p* = 0.016].

Opposite to that observed with delta power, on day 1 only high dose kratom significantly decreased theta power across the 30-min testing period when compared to low dose kratom or saline controls (*p* < 0.001, Figure 2E, left panel). On day 7, a baseline suppression in theta power was evident in the high dose kratom group compared to both the low dose kratom (*p* = 0.038) and control group (*p* = 0.013), an effect strengthened after the final injection of high dose kratom that was maintained (*p* = 0.003 versus controls, Figure 2E, right panel). Reduced theta power was also evident in the low dose group, however this effect was short-lived, only evident at 5 min post-injection (*p* = 0.001, compared to saline controls) [Time: F(5,105) = 3.6, *p* = 0.005; Time × Treatment: F(10,105) = 3.8, *p* < 0.001; Treatment: F(2,21) = 10.4, *p* = 0.001]. In the NAc on day 1 and day 7, there were no significant effects of treatment in the beta and low gamma frequency bands in response to either dose of kratom (Figures 2F,G). Similarly, there were no observed effects in the high gamma frequency band on day 1 (Figure 2H, left panel). However, on day 7, baseline recordings showed that animals that had received repeated administration of high dose kratom had lower baseline high gamma power compared to the low dose group (*p* = 0.006) or saline controls (*p* = 0.002) (Figure 2H, right panel). Post-injection, this decrease in high gamma power was maintained throughout the recording period such that high dose kratom reduced high gamma power compared to both the lower dose (*p* = 0.03) of kratom and saline controls (*p* = 0.006) [Treatment: F(2,22) = 6.4, *p* = 0.007]. Together these findings indicate significant effects of acute and chronic high dose kratom on NAc low frequency power, with an additional suppression of high gamma power selectively with repeated administration.

### Prefrontal Cortex

**FIGURE 3:**
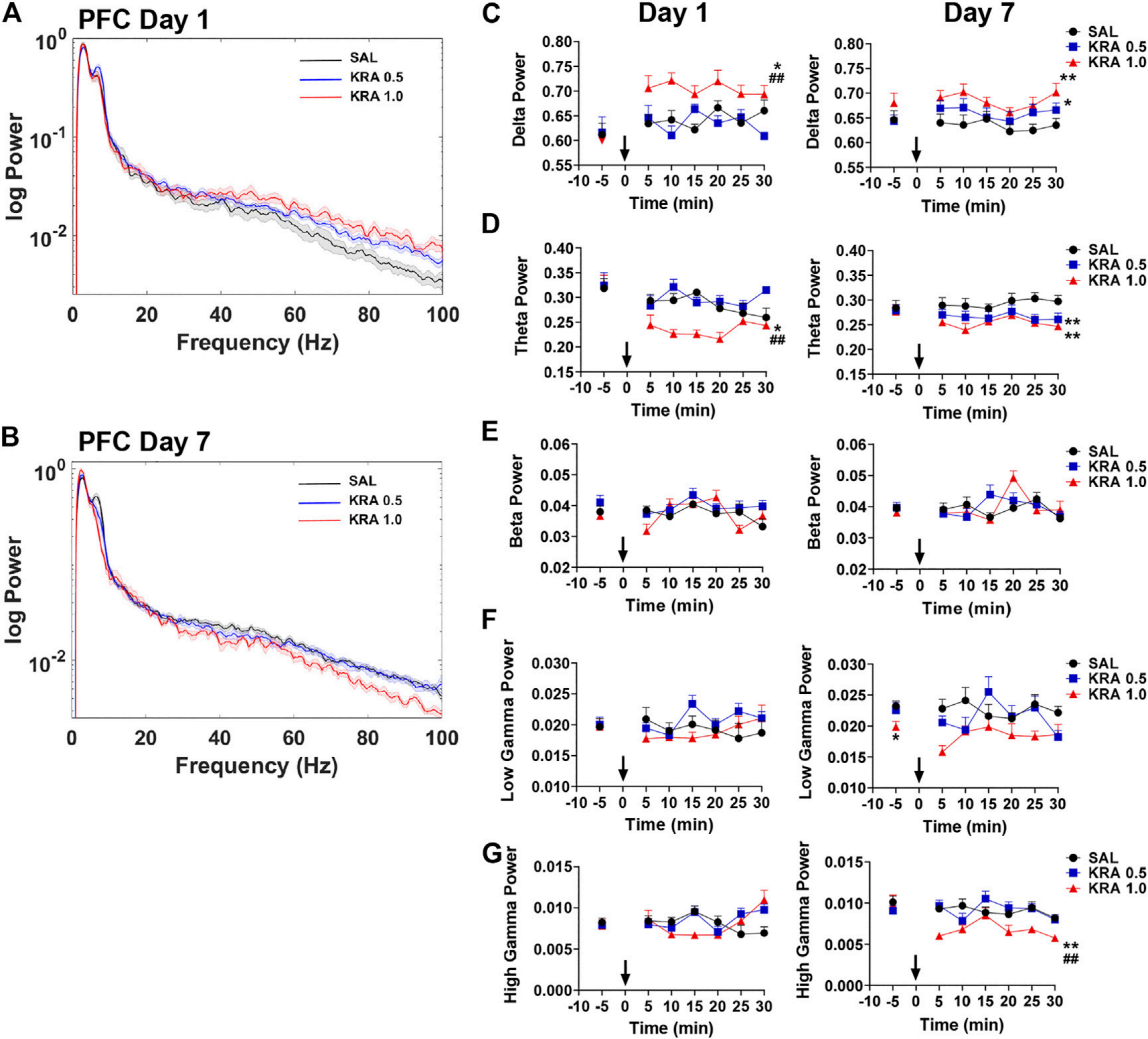
Time course of dose-dependent effects of kratom on PFC spectral power. **(A,B)** Representative power spectra showing dose-dependent (0, 0.5, 1.0 mg/kg) effects of kratom 30 min post-injection on day 1 and day 7. **(C)** High dose kratom increased delta power on day 1 and day 7 whereas low dose kratom increased delta power on day 7. **(D)** Reduced theta power in response to only high dose kratom on day 1. On day 7, decreased theta power was shown in response to both doses of kratom. **(E,F)** No drug-induced changes were observed in beta or low gamma power on day 1 or day 7. **(G)** On day 1, kratom had no effects on high gamma power. Reduced high gamma power was, however, evident on day 7 in response to high dose kratom. *N* = 7–10 rats per group, with 2 electrodes/rat. Curves are represented as means with jackknife estimates of sem depicted by the shaded areas. Bars represent mean ± sem. *p < 0.05, **p < 0.01 compared to saline control rats. ^##^ *p* < 0.01 compared to low dose kratom treated rats.

In the PFC, representative power spectra showing the effects of kratom 30 min post-injection on day 1 and day 7 are shown (Figures 3A,B). In this region, the repeated measures ANOVA revealed a significant effect of Treatment on delta power on both day 1 and day 7 (Figure 3C) [Day 1: Treatment: F(2,26) = 10.0, *p* = 0.001; Day 7: Treatment: F(2,20) = 8.0, *p* = 0.003]. Overall, only the high dose kratom increased delta power on day 1, relative to the low dose kratom (*p* = 0.001) and saline controls (*p* = 0.012) (Figure 3C, left panel). However, on day 7, both low and high dose kratom were found to significantly increase delta power across 30 min (*p* = 0.019 and *p* = 0.003, respectively) (Figure 3C, right panel). Opposite to the observed delta power changes, a significant decrease overall in theta power was induced by high dose kratom on day 1 in the PFC compared to low dose kratom (*p* < 0.001) and controls (*p* = 0.014), with no effects of low dose kratom (Figure 3D, left panel) [Time × Treatment: F(10,140) = 2.0, *p* = 0.036; Treatment: F(2,28) = 12.6, *p* < 0.001]. On day 7, however, both low (*p* < 0.001) and high (*p* = 0.002) dose kratom suppressed theta power across the recording period (Figure 3D, right panel) [Treatment: F(2,23) = 11.4, *p* < 0.001]. There were no effects of kratom administration on spectral power in the beta frequency band on either day (Figure 3E). Similarly, no observed drug effects in low or high gamma power were evident on day 1 (Figures 3F,G, left panels). However, baseline data taken prior to the day 7 injection showed that animals had received prior treatment with high dose kratom had suppressed low gamma power (*p* = 0.029) compared to saline controls (Figure 3F, right panel). Following the day 7 injection, high dose kratom suppressed high gamma power compared to both low dose kratom and control groups (*p* < 0.01) (Figure 3G, right panel) [Treatment: F(2,25) = 7.3, *p* = 0.003]. Overall, similar to that observed in NAc, these findings demonstrate prominent effects of kratom in the low frequency range in the PFC, with additional effects to suppress high gamma power.

### Cingulate Cortex

**FIGURE 4:**
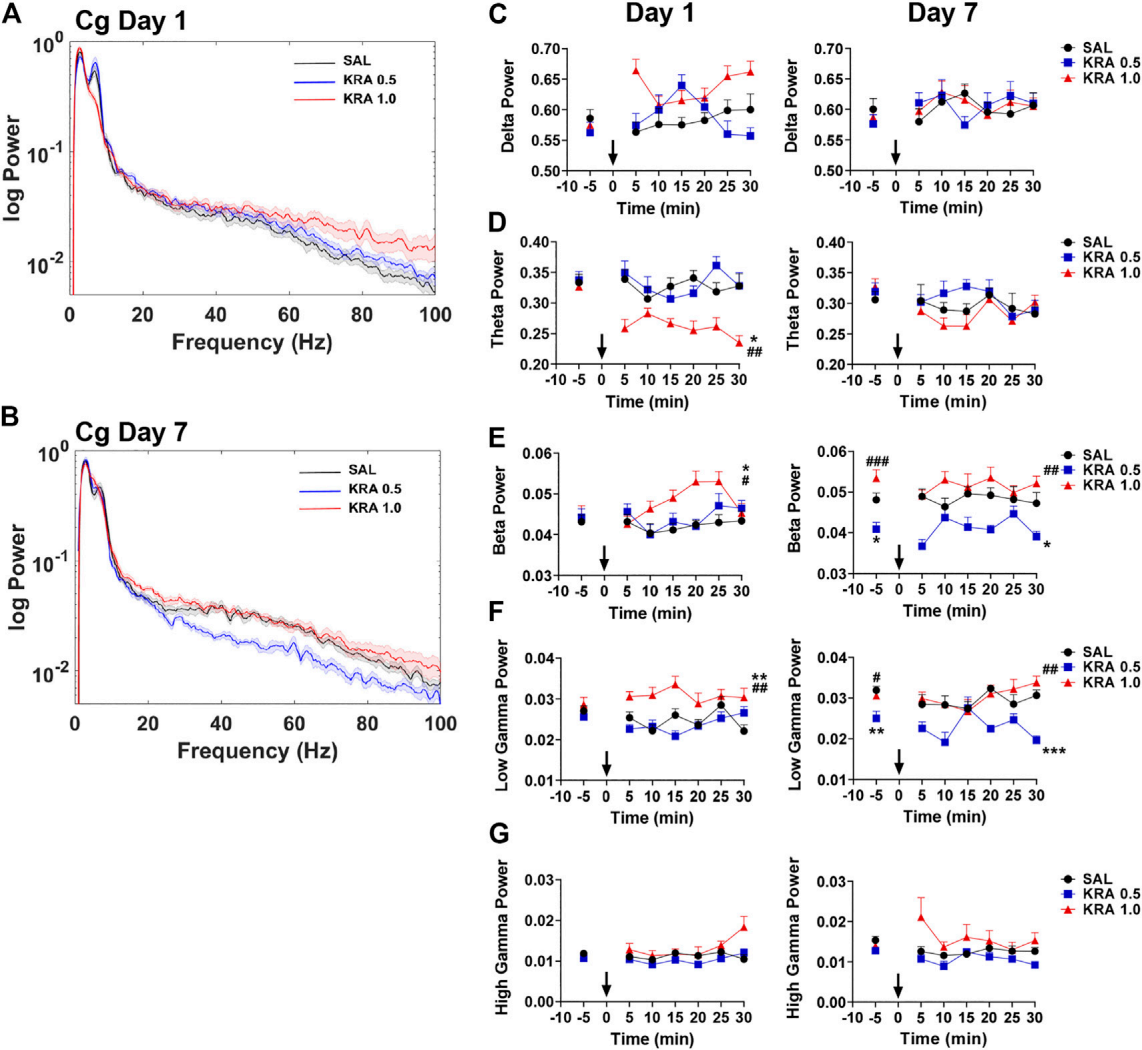
Time course of dose-dependent effects of kratom on Cg spectral power. **(A,B)** Representative power spectra showing dose-dependent (0, 0.5, 1.0 mg/kg) effects of kratom 30 min post-injection on day 1 and day 7. **(C)** No kratom-induced changes in delta power were evident on day 1 or day 7 in the Cg. **(D)** High dose kratom supressed theta power in on day 1 with no changes evident on day 7. **(E)** High dose kratom increased beta power on day 1. On day 7, reduced baseline beta power was observed in response to low dose kratom, and this change persisted following the final injection. **(F)** High dose kratom elevated low gamma power on day 1. Low dose kratom decreased baseline low gamma power on day 7 and this effect was maintained after the last injection. **(G)** No effects of kratom treatment were observed in the high gamma frequency band. *N* = 7–10 rats per group, with 2 electrodes/rat. Curves are represented as means with jackknife estimates of sem depicted by the shaded areas. Bars represent mean ± sem. *p < 0.05, **p < 0.01, ***p < 0.001 compared to saline control rats. ^##^ *p* < 0.01, ^###^ *p* < 0.01 compared to low dose kratom treated rats.

Kratom-induced changes in neural oscillatory power in the Cg were next evaluated (Figure 4), with representative power spectra at 30 min displayed in Figures 4A,B. On the first day of testing, a repeated measures ANOVA revealed a significant interaction between Time and Treatment in delta power within the Cg [delta: F(10,115) = 2.2, *p* = 0.022]. Overall, no significant group differences were found on either day (Figure 4C). For the theta frequency band, high dose kratom induced a significant decrease in oscillatory power on day 1, compared to both low (*p* = 0.007) dose kratom and saline controls (*p* = 0.011) [Treatment: F(2,23) = 6.5, *p* = 0.006], but not on day 7 (Figure 4D, right panel). When beta power was examined, significant effects of Treatment were observed on both days [beta day 1: Treatment: F(2,23) = 5.2, *p* = 0.014; beta day 7: Treatment: F(2,20) = 10.9, *p* < 0.001]. On day 1, high dose kratom significantly increased beta power compared to low dose kratom (*p* = 0.012) and controls (*p* = 0.028) (Figure 4E, left panel). On day 7, baseline differences in beta power were observed, such that animals that received low dose kratom repeatedly prior to the final day of testing had a significantly reduced baseline beta power (*p* = 0.019). Following the final injection, this decrease in beta power persisted across the 30 min testing period (*p* = 0.017) (Figure 4E, right panel). As well, in the Cg, following an acute injection of high dose kratom, a significant increase in low gamma power was observed (Figure 4F, left panel), relative to low dose kratom (*p* = 0.001) and saline control (*p* = 0.004) groups [Treatment: F(2,21) = 10.6, *p* = 0.001]. On day 7, low dose kratom decreased low gamma power baseline measures (*p* = 0.008) and, following the final injection, this effect was maintained (*p* < 0.001) (Figure 4F, right panel) [Treatment: F(2,20) = 11.6, *p* < 0.001]. Overall, in the high gamma frequency band no significant group differences were found on day 1 or day 7 (Figure 4G [Time × Treatment: F(10,115) = 3.3, *p* = 0.001]. These findings indicate that, unlike NAc and PFC, the effects of kratom in the Cg appear restricted to the theta and low gamma frequency bands.

### Ventral Tegmental Area

**FIGURE 5:**
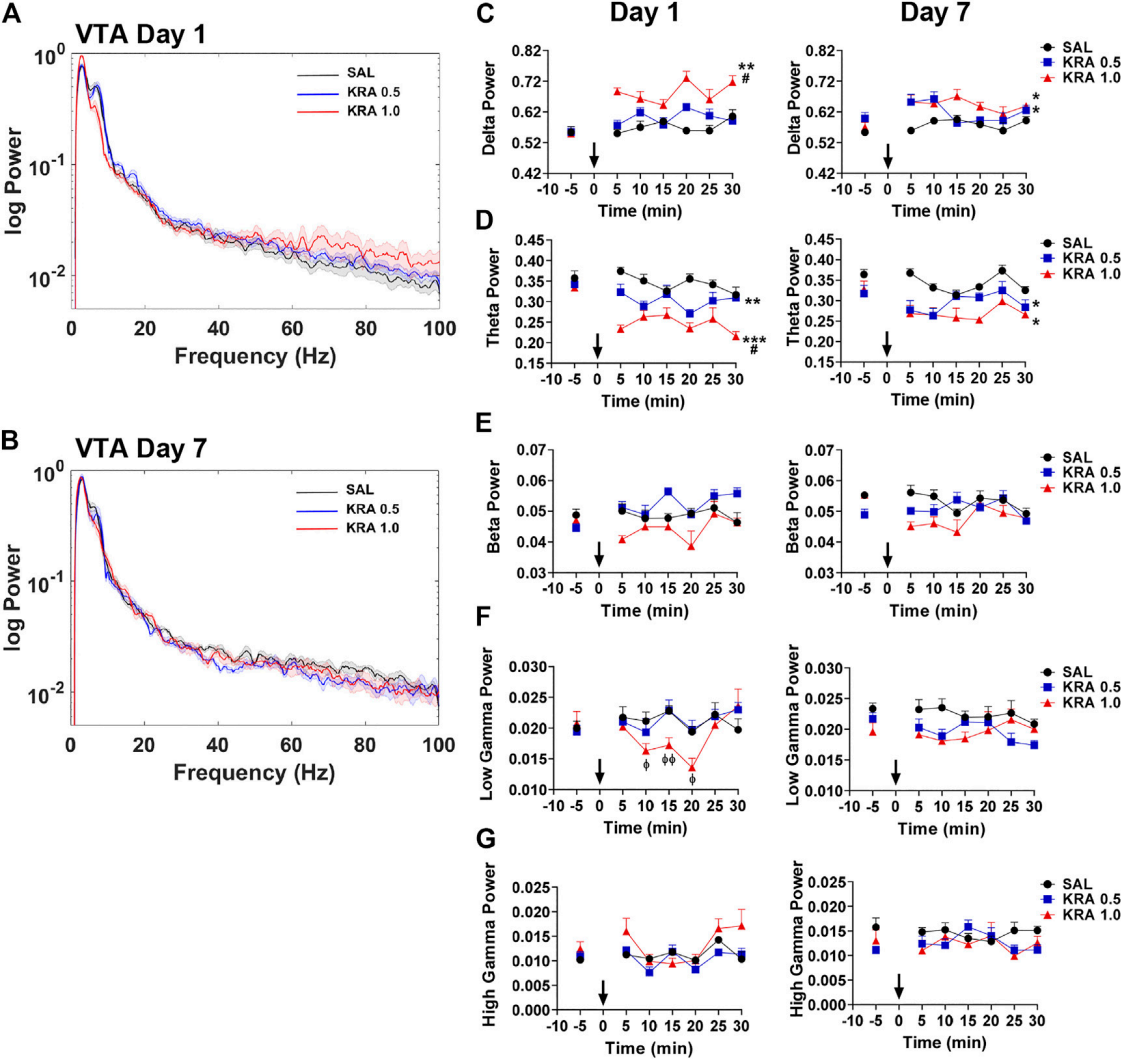
Time course of dose-dependent effects of kratom on VTA spectral power. **(A,B)** Representative power spectra showing dose-dependent (0, 0.5, 1.0 mg/kg) effects of kratom 30 min post-injection on day 1 and day 7. **(C)** On day 1, high dose kratom increased delta power whereas on day 7, an elevation in power was induced by both doses of kratom. **(D)** On both day 1 and day 7, both doses of kratom resulted in a reduction in theta power. **(E)** No drug-induced changes in beta power were evident. **(F)** A transient decrease in the low gamma power in response to only high dose kratom on day 1 with no effects on day 7 **(G)** No effects of kratom treatment on high gamma power on day 1 or day 7. *N* = 7–10 rats per group, with 2 electrodes/rat. Curves are represented as means with jackknife estimates of sem depicted by the shaded areas. Bars represent mean ± sem. *p < 0.05, **p < 0.01, ***p < 0.001 compared to saline control rats. ^#^ *p* < 0.05 compared to low dose kratom treated rats.

Alterations in VTA spectral power 30 min following acute or repeated kratom injections are depicted in Figures 5A,B. Only an acute injection of high dose kratom resulted in elevated delta power compared to low dose kratom (*p* = 0.015) and control (*p* = 0.001) groups (Figure 2, Figure5C, left panel) [Time x Treatment: F(10,115) = 1.9, *p* = 0.045; Treatment: F(2,23) = 9.9, *p* = 0.001]. On the final day of testing both high (*p* = 0.018) and low (*p* = 0.041) dose kratom elevated delta power (Figure 5C, right panel) [Time: F(5,100) = 3.5, *p* = 0.006; Time x Treatment: F(10,100) = 2.0, *p* = 0.038; Treatment: F(2,20) = 5.0, *p* = 0.018]. Similar to the observed effects of acute high dose kratom on theta power in the NAc, PFC and Cg, a significant decrease in VTA theta power was shown (*p* < 0.001) (Figure 5D, left panel). A similar, albeit less robust, effect was observed in response to low dose kratom a (*p* = 0.006) [Treatment: F(2,26) = 16.4, *p* < 0.001]. On day 7, these dose-dependent effects were maintained (Figure 5D, right panel) [Time: F(5,105) = 5.2, *p* < 0.001; Treatment: F(2,21) = 5.2, *p* = 0.014]. There were no observed changes in beta power in response to kratom administration on either day of testing (Figure 5E). Repeated measures ANOVA revealed time-dependent changes in low gamma power in response to high dose kratom whereby there was a transient decrease in low gamma power that normalized by the end of the 30 min (Figure 5F, left panel) [Time: F(5,115) = 3.7, *p* = 0.004; Time x Treatment: F(10,115) = 4.4, *p* < 0.001]. There were no group differences in low gamma power on day 7. For high gamma power, no drug effects were evident on day 1 or 7, although a Treatment effect was evident (Figure 5G, right panel) [Treatment: F(2,21) = 5.7, *p* = 0.011]. Thus, the effects of kratom in the VTA were restricted to the low frequency bands delta and theta.

### Coherence

**FIGURE 6:**
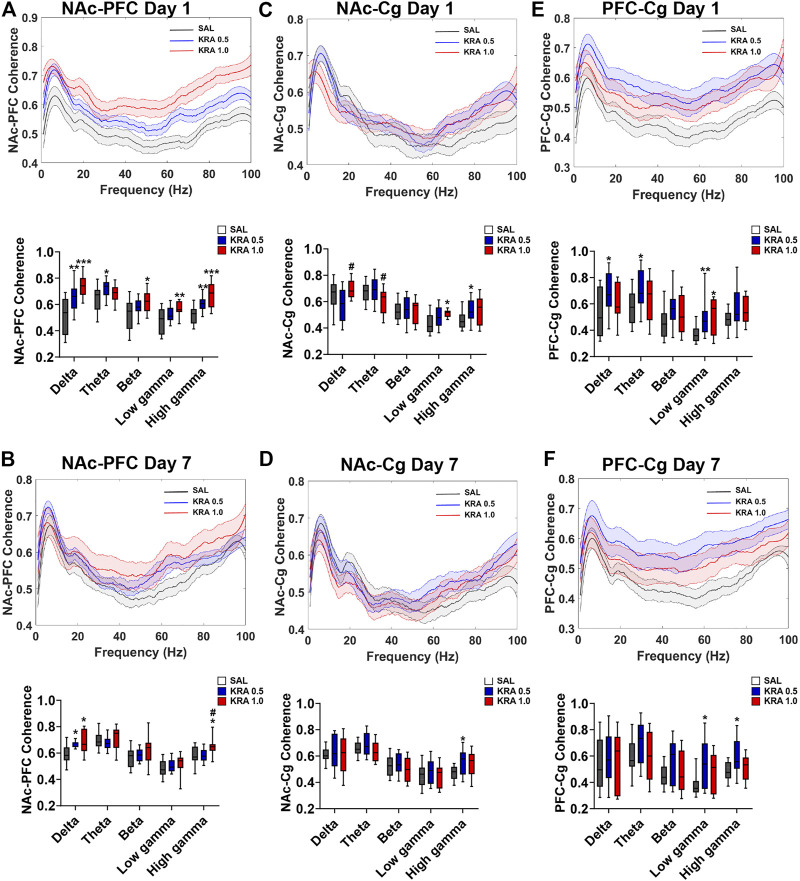
Dose-dependent effects of acute and repeated kratom administration on NAc-PFC, NAc-Cg, and PFC-Cg coherence. Coherence across frequencies and quantification showing the effect of acute and repeated low (0.5 mg/kg) or high (1 mg/kg) dose kratom 30 min post-injection. Data shown were taken from 30 s epochs. **(A)** On day 1, both doses of kratom increased NAc-PFC delta and high gamma coherence. Low dose kratom increased theta coherence and high dose kratom increased beta and low gamma coherence. **(B)** On day 7, both doses of kratom increased delta coherence whereas only high dose kratom induced an increase in high gamma coherence. **(C)** On day 1, high dose kratom increased low gamma NAc-Cg coherence whereas low dose kratom increased high gamma coherence. **(D)** NAc-Cg coherence was increased in the high gamma frequency band only in response to low dose kratom on day 7. **(E)** Low dose kratom increased low frequency and low gamma coherence between the PFC-Cg. High dose kratom also increased coherence in the low gamma frequency band on day 1. **(F)** On day 7, only low dose kratom elevated PFC-Cg low and high gamma coherence. *N* = 7–10 rats per group, with 2 electrodes/rat. Curves are represented as means with jackknife estimates of sem depicted by the shaded areas. Quantification of coherence are represented as boxplots with min/max values. *p < 0.05, **p < 0.01, ***p < 0.001 compared to saline control rats. ^#^ *p* < 0.05 compared to low dose kratom treated rats.

**FIGURE 7:**
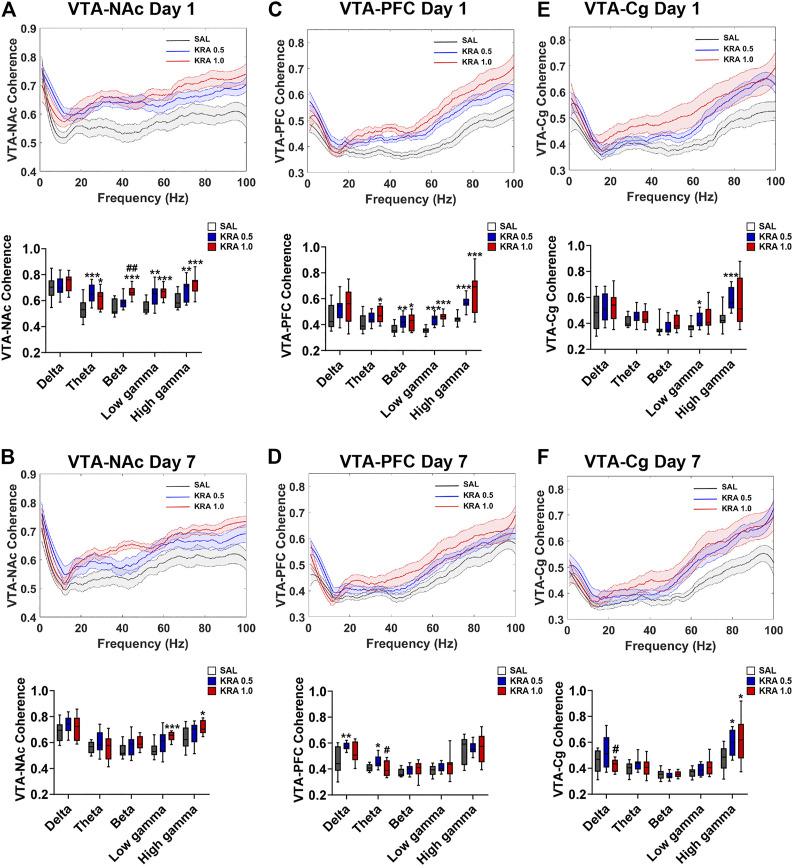
Dose-dependent effects of acute and repeated kratom administration on VTA-NAc, VTA-PFC, and VTA-Cg coherence. Coherence across frequencies and quantification showing the effect of acute and repeated low (0.5 mg/kg) or high (1 mg/kg) dose kratom 30 min post-injection. Data shown were taken from 30 s epochs. **(A)** High dose kratom increased VTA-NAc beta coherence whereas theta, low gamma and high gamma coherence was increased by both doses of kratom. **(B)** On day 7, only high dose kratom increased low and high gamma coherence between VTA-NAc. **(C)** VTA-PFC coherence was increased in the high frequency bands in response to both doses of kratom. In the theta band, only high dose kratom increased coherence on day 1. **(D)** On day 7, repeated injections of low dose kratom increased VTA-PFC low frequency coherence. **(E)** An acute injection of low dose kratom elevated VTA-Cg low and high gamma coherence. **(F)** Repeated injections of both doses of kratom increased high gamma coherence between VTA-Cg. *N* = 7–10 rats per group, with 2 electrodes/rat. Curves are represented as means with jackknife estimates of sem depicted by the shaded areas. Quantification of coherence are represented as boxplots with min/max values. *p < 0.05, **p < 0.01, ***p < 0.001 compared to saline control rats. ^#^ *p* < 0.05, ^##^ *p* < 0.01 compared to low dose kratom treated rats.

To evaluate the effect of both acute and repeated kratom administration on inter-regional communication, coherence was assessed on the first and final day of testing, just prior to behavioural testing (Figures 6, 7). On the first day of testing, a Main Effect of Treatment in the NAc-PFC was evident in all frequencies (Figure 6A) [delta: F(2,41) = 13.2, *p* < 0.001; theta: F(2,40) = 4.3, *p* = 0.02; beta: F(2,43) = 4.1, *p* = 0.02; low gamma: F(2,42) = 5.6, *p* = 0.007; high gamma: F(2,44) = 15.0, *p* < 0.001]. In the delta and high gamma bands, both low (*p* = 0.005) and high (*p* < 0.001) doses of kratom resulted in elevated coherence in comparison to the control animals. In the theta frequency, only low dose kratom (*p* = 0.015) increased coherence relative to controls. On day 7, this elevated NAc-PFC coherence was evident only in the delta and high gamma frequencies (Figure 6B) [delta: F(2,32) = 6.0, *p* = 0.006; high gamma: F(2,37) = 4.3, *p* = 0.02]. However, while both low (*p* = 0.015) and high (*p* = 0.03) doses of kratom increased coherence in the delta band, only the high dose increased high gamma coherence (*p* = 0.04).

Upon examination of NAc-Cg coherence, a significant Treatment Effect of kratom was found in the low frequencies and in the gamma frequencies on the first day of testing (Figure 6C). [delta: F(2,39) = 4.9, *p* = 0.013; theta: F(2,43) = 3.4, *p* = 0.04; low gamma: F(2,39) = 4.0, *p* = 0.027; high gamma: F(2,41) = 3.4, *p* = 0.04). On day 1, low dose (*p* = 0.014) and high dose (*p* = 0.014) kratom increased coherence in high and low gamma bands, respectively compared to controls. This increased coherence in the high gamma band following administration of low dose kratom was maintained on day 7 (*p* = 0.02) (Figure 6D) [Treatment Effect in high gamma on day 7; F(2,39) = 3.5,*p* = 0.038]. When PFC-Cg coherence was examined, a significant kratom Treatment effect on day 1 was found in the delta and theta bands, as well as the low gamma frequency (Figure 6E) [delta: F(2,42) = 4.8, *p* = 0.014; theta: F(2,45) = 3.5, *p* = 0.04; low gamma: F(2,40) = 4.7, *p* = 0.015]. In the low gamma frequency, both low (*p* = 0.007) and high (*p* = 0.016) doses of kratom increased coherence. On day 7, the increase in low gamma coherence as a result of kratom treatment persisted, but only for the low dose (*p* = 0.014) (Figure 6F) [Treatment Effect in low gamma on day 7: F(2,38) = 3.8, *p* = 0.03].

A significant effect of Treatment in both VTA-NAc coherence and VTA-PFC coherence on day 1 was found in the theta, beta, low gamma and high gamma frequencies [VTA-NAc: theta: F(2,41) = 10.2, *p* < 0.001; beta: F(2,35) = 18.6, *p* < 0.001; low gamma: F(2,38) = 11.3, *p* < 0.001; high gamma: F(2,40) = 10.2, *p* < 0.001][VTA-PFC: theta: F(2,40) = 4.0, *p* = 0.025; beta: F(2,42) = 6.1, *p* = 0.005; low gamma: F(2,39) = 28.6, *p* < 0.001; high gamma: F(2,38) = 15.9, *p* < 0.001]. Examining VTA-NAc coherence (Figure 7A) on day 1, both low (*p* < 0.001) and high (*p* = 0.017) doses of kratom increased theta coherence. In the beta band, there was a significant increase in coherence in response to the high dose kratom, relative to the low dose group (*p* = 0.001) and controls (*p* < 0.001). When low gamma coherence was assessed, both low and high dose kratom (*p* = 0.002, *p* < 0.001 respectively) increased coherence. Similarly, an increase in high gamma coherence was observed in response to both doses of kratom (*p* = 0.009 for low dose and *p* < 0.001 for high dose kratom). An effect of Treatment in VTA-NAc coherence persisted at day 7 in the gamma frequency bands (Figure 7B) [low gamma: F(2,36) = 7.0, *p* = 0.003; high gamma: F(2,360) = 3.9, *p* = 0.028]. Finally, when assessing VTA-PFC coherence (Figure 7C) on day 1 in the beta frequency, there was an increase in beta coherence in response to the low (*p* = 0.002) and high (*p* = 0.039) doses of kratom. In the low gamma band, both kratom doses increased coherence (*p* < 0.001 and *p* < 0.001 respectively). Similarly, a significant increase in high gamma coherence was observed in response to both doses of kratom (*p* < 0.001 for both doses). The kratom Treatment effects that were apparent on day 1 in VTA-PFC coherence were not maintained following the repeated administration of kratom (Figure 7D).

A Treatment effect in VTA-Cg coherence was evident in the low and high gamma frequency bands following an acute injection of kratom (Figure 7E) [low gamma: F(2,42) = 3.6, *p* = 0.036; high gamma: F(2,39) = 5.5, *p* = 0.008]. In the high gamma band specifically, only the low dose of kratom increased coherence (*p* < 0.001). On day 7, there was again an effect of Treatment in the high gamma band [F(2,39) = 4.4, *p* = 0.019] with increased coherence following administration of low dose kratom (*p* = 0.012) (Figure 7F).

### Behaviour

**FIGURE 8:**
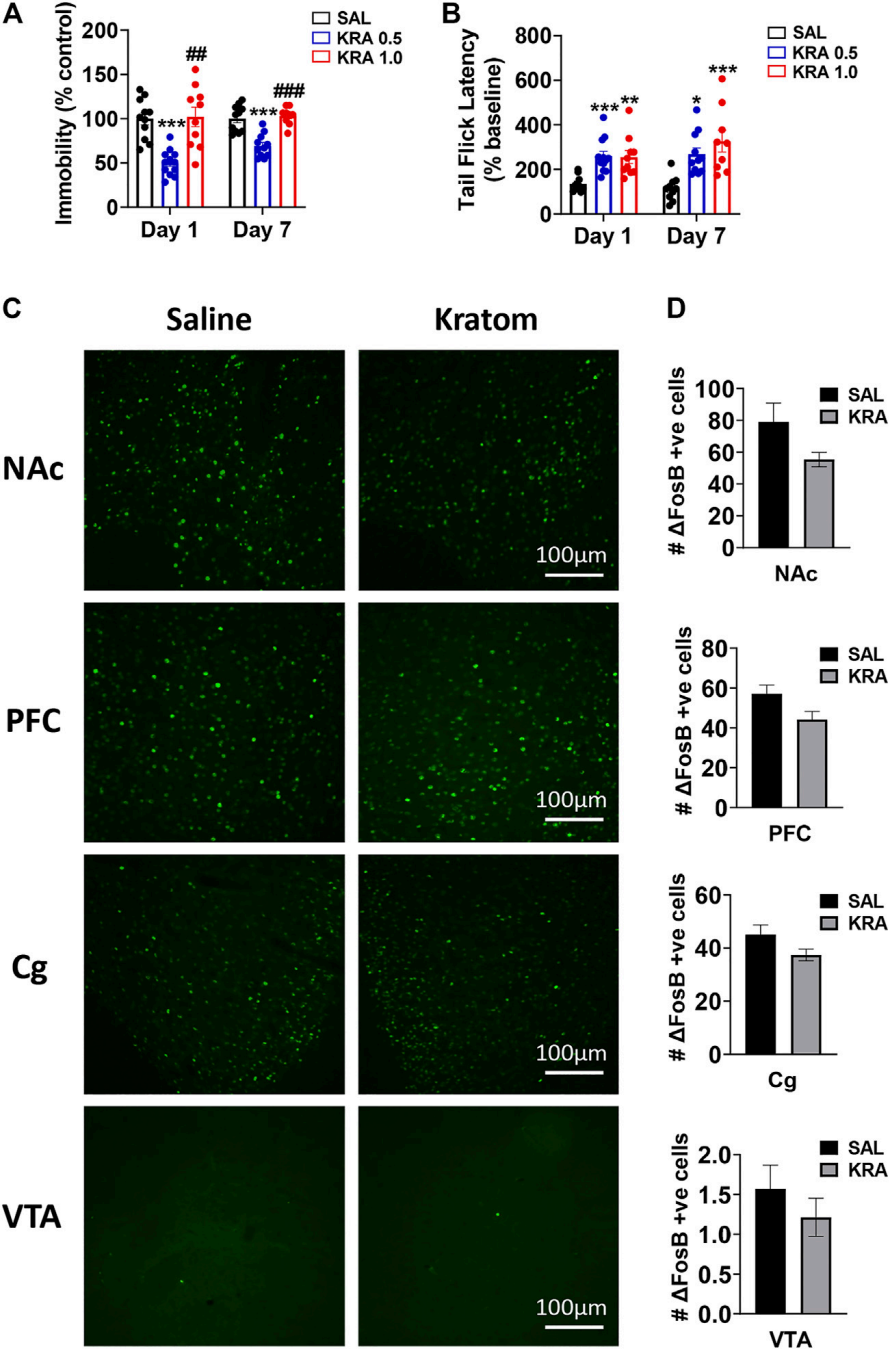
Effects of kratom on behaviour and ΔFosB expression in rats. **(A)** Low (0.5 mg/kg) dose, but not high (1 mg/kg) dose kratom significantly reduced immobility time in the FST 30 min post-injection on both day 1 and day 7. **(B)** Both doses of kratom significantly increased tail-flick latencies compared to pre-drug baseline latencies 40 min post-acute and repeated injections. *N* = 10–13 rats per group. **(C,D)** Representative images and quantification depicting no change in ΔFosB expression in response to high dose kratom in any brain region. *N* = 7-8 rats per group. Bars represent mean ± sem. *p < 0.05, **p < 0.01, ***p < 0.001 compared to saline control rats. ^##^ *p* < 0.01, ^###^ *p* < 0.001 compared to low dose kratom treated rats.

To investigate the potential antidepressant-like and analgesic effects of kratom, dose-dependent drug effects were evaluated first in the FST, followed by the tail-flick test, immediately after the LFP recordings (Figure 8A). Following a single injection of kratom, we found that low dose kratom significantly reduced immobility in the FST compared with saline controls (88.8 ± 25.5 *versus* 176.8 ± 40.1, *p* < 0.001) (F(2,30) = 16.3, *p* < 0.001). This selective decrease in immobility was again apparent following daily administration of low dose kratom for 7 days (165.4 ± 31.3 *versus* 239.1 ± 35.6, *p* < 0.001) (F(2,28) = 22.0, *p* < 0.001). There were no effects of high dose kratom on immobility time in the FST. However, it should be noted that the variability of the high dose group on day 1 was much greater than that observed on day 7. Further, between day 1 and day 7, there was an overall increase in FST immobility across all groups (approximately 52%) that was likely representative of learned behaviour. However, the direction and magnitude of group differences were maintained between both days of testing. Next, we evaluated the analgesic efficacy of both doses of kratom using the tail-flick test at 40 min post-injection (Figure 8B). On both days of testing, animals treated with either dose of kratom showed increased tail-flick latencies, when compared with baseline values. On day 1, significant analgesic effects were observed for both low dose (*p* < 0.001) and high dose (*p* = 0.003) kratom (F(2,30) = 10.5, *p* < 0.001). Following repeated administration, both doses again displayed significant analgesic effects (*p* = 0.011 for low dose kratom, *p* < 0.001 for high dose kratom) (F(2,28) = 10.5, *p* < 0.001).

### ΔFosB Expression

There has been some evidence to suggest that 7-HMG, one of the compounds found in kratom, has addictive properties (Hemby et al., 2019). As the transcription factor ΔFosB has been suggested as a molecular switch for addiction (Nestler et al., 2001), the impact of repeated treatment with high dose kratom on expression of ΔFosB was next evaluated (Figures 8C,D). The high dose was chosen as the mean analgesic response was slightly higher in this group, with some animals showing the strongest analgesic responses (up to 600% increase in tail-flick latency from baseline). The ANOVA revealed a significant Main Effect of Treatment [F(3,48) = 9.6, *p* = 0.003]. Within each region there were no significant drug effects although trends towards reduced ΔFosB expression were evident that were strongest in the PFC (*p* = 0.057, Figure 8D).

## Discussion

In the present study, we sought to evaluate the dose-dependent analgesic and antidepressant-like effects induced by the kratom alkaloid extract in rats, and to elucidate changes in neural oscillatory patterns following acute and repeated drug administration. We found that only the low dose of kratom resulted in antidepressant-like effects, reducing the immobility time in the FST after both acute and repeated administration. In addition, both doses of kratom demonstrated analgesic properties in the tail-flick test upon initial administration as well as following repeated dosing. These behavioural effects were accompanied by a dose-and region-specific elevation in delta power and the suppression of theta and high gamma power. Further, enhanced coherence between all brain regions in response to both doses of kratom were shown on the first and final day of testing, with the most robust effects observed in the NAc-PFC, VTA-NAc, and VTA-Cg pathways. No significant changes in the expression of the addiction marker ΔFosB were evident in any of the brain regions following repeated administration of the high dose of kratom.

Taken together, the analgesic activity and antidepressant potential of kratom alkaloids that were observed in this study support anecdotal evidence surrounding the use of the plant among mainstream kratom enthusiasts—indeed, the consumption of kratom as a self-treatment strategy for the relief of acute and chronic pain are on the rise, especially in the United States (Kruegel and Grundmann, 2018; Veltri and Grundmann, 2019; Palamar, 2021). Our findings also underscore two additional important points, the first being that the alkaloid composition in kratom can vary depending on the growth conditions. For example, although mitragynine is often reported to be the most abundant alkaloid in kratom, our evidence shows that this is not always the case. Indeed, we showed that the alkaloids 3-isoajmalicine and speciogynine were twice as abundant than mitragynine in our samples. The second point is that the biological effects of kratom as a whole may be different then that of its innate chemical constituents, a noteworthy consideration as the pharmacology of kratom has traditionally focused on the individual alkaloids that typically accumulate within the plant. Accordingly, and with mixed results, these studies have often not considered how the suite of kratom alkaloids behave in a biological system. This latter point is especially relevant considering that dried kratom leaves or a decoction of the alkaloids therein is the primary mode of consumption among end users.

### Oscillatory Changes Accompanying the Analgesic Effects of Kratom

To date, a limited number of experiments conducted thus far have demonstrated the analgesic efficacy of the entire kratom extract, with all of its alkaloids present. Therefore, we evaluated the analgesic effects of two doses of kratom in the tail-flick test, a behavioural test used to assess heat-evoked pain in animals. We found that both the low and high doses of kratom increased tail-flick latencies on the first and final day of injections, suggesting analgesic effects. These findings are consistent with studies that also have investigated the effect of kratom extracts on the behavioural output in the tail-flick test (Sabetghadam et al., 2010), as well as in the hot plate test, another behavioural measure of analgesia in rodents (Reanmongkol et al., 2007). It is important to note however, that one study reported analgesic effects in the hot plate test but not the tail-flick test in response to kratom (Reanmongkol et al., 2007). This may be due to differences in extract preparation and origin of the kratom leaf (Sabetghadam et al., 2010). Further, some authors have postulated that a reason for this difference could be due to the different components involved in both the hot plate and tail-flick test whereby supraspinal pathways and spinal pathways are involved, respectively (Matsumoto et al., 2004; Reanmongkol et al., 2007).

The present findings demonstrated oscillatory changes within the VTA and NAc in response to both doses of kratom, two regions of the mesolimbic dopamine system, a pathway that has been found to be activated in response to acute pain as well as pain relief (Borsook et al., 2016), and regions that play important roles in mediating the rewarding and analgesic effects of opioids (Wise, 1989; Harris and Peng, 2020). These regions in particular are suggested to be involved in pain processing, as the offset of pain has been found to be rewarding (Borsook et al., 2016; Harris and Peng, 2020). This idea is supported through clinical neuroimaging studies conducted in individuals suffering from chronic pain (Wood et al., 2007) as well as in individuals presented with noxious stimuli (Becerra et al., 2001; Becerra and Borsook, 2008) where activation of the mesolimbic network is observed. Therefore, this pathway is considered an essential target for the treatment of pain as its activation is believed to induce analgesic effects and may modulate the effectiveness of analgesic medications (Mitsi and Zachariou, 2016; Taylor et al., 2016; Kami et al., 2018).

Overall, in the NAc and VTA kratom administration induced an increase in delta power that was concomitant with a reduction in theta power. Interestingly, these findings are similar to another preclinical study using rats that found reduced theta power in the NAc and increased delta power in the VTA following morphine administration (Ahmadi Soleimani et al., 2018). Morphine is an established analgesic that is similar to the kratom alkaloids mitragynine and 7-HMG, in that it binds to the MOR to exert its effects (Ream and Michael, 2011; Kruegel et al., 2016). Therefore, it is possible that the similarities in the drug-induced electrophysiological patterns may play a role in the reported analgesic properties of these compounds. Our observed changes in NAc, however, were not in agreement with a study conducted by Cheaha et al. (2015) that showed an absence of oscillatory changes in NAc of mice when administered 80 mg/kg of an alkaloid enriched kratom extract. Aside from the species used in the studies, this discrepancy likely results from differences in the extract used. Specifically, whereas the extract from Cheaha et al. (2015) was enriched with mitragynine, the extract used herein showed significant levels of other alkaloids, two of which that were in greater abundance than mitragynine. Indeed, we have previously shown that synthetic mitragynine has no effects on NAc oscillations (Thériault et al., 2020). Differences in the composition of the extract are also exemplified by the dose used, with their dose as much as 160 times higher than the one used in the present study. Together these findings do highlight, however, that differences in plant composition potentially produce discrete and significant differences on brain function.

Although we did see changes in delta and theta oscillations within the mesolimbic pathway, our observed effects were not always found with both doses on each assessment day, despite analgesia being evident upon acute and at the end of repeated administration of kratom. This suggests that while these oscillatory changes could reflect alterations in the activity of the mesolimbic pathway, it may be that analgesia is not specifically coupled to these region and frequency-specific oscillations. We also noted that there were long lasting drug-induced changes found in specific frequencies prior to the final injection that occurred in the NAc, Cg and PFC, but not in the VTA, reflecting region-dependent differences in response duration that lasted at least 24 h. Such long lasting functional changes are notable as, to our knowledge, such prolonged effects are not seen with morphine, which has a half-life of approximately 2 h in rodents (Emery et al., 2017). Of critical importance, there is conflicting evidence as to whether mitragynine has agonist activity at murine or rat MORs (Kruegel et al., 2016; Obeng et al., 2021) suggesting that the observed kratom-induced effects may be mediated by other receptors, by other alkaloids in the extract, or *via* mitragynine metabolites. Indeed, 7-HMG does have agonist properties at rodent MORs (Kruegel et al., 2016; Obeng et al., 2021). Furthermore, the extract used in the present study contained substantial quantities of speciogynine and 3-isoajmalicine. Although little is known about the pharmacological and physiological impacts of these compounds in brain, pharmacological activity may be MOR-independent as they do not appear to have pharmacological activity at MORs (Kruegel et al., 2016). Added to this speciogynine, as well as other alkaloids in the extract such as corynantheidine, speciocilliatine, and paynantheine, have been demonstrated to have moderate or potent inhibitory effects on CYP enzymes (Kamble et al., 2020), which may contribute to increased duration of effects due to reduced metabolism. With respect to VTA-NAc coherence, elevated activity was observed in the high frequency bands following an acute injection of either dose of kratom, with the effects also present after 7 days selectively with the high dose. This action appears similar to that of morphine, with a previous study in mice showing a morphine-induced increase in VTA-NAc gamma coherence (Reakkamnuan et al., 2017). Clinical electroencephalogram (EEG) studies have previously reported that the perception of pain may be associated with gamma rhythms, and that the disruption of these rhythms may contribute to analgesic effects (Whittington et al., 1998; Croft et al., 2002).

Perhaps one of the most interesting findings was the observation of increased delta coherence between the NAc-PFC in response to both doses of kratom following acute and repeated injections. The projection from the PFC to the NAc has been reported to be implicated in the regulation of pain (Baliki et al., 2010). Preclinical studies have also demonstrated the involvement of this pathway in the modulation of pain through the inactivation or activation of NAc-PFC connections (Lee et al., 2015; Martinez et al., 2017; Zhou et al., 2018). Specifically, one of these studies demonstrated that activation of NAc-PFC projections through optogenetics resulted in pain relief when animals were subjected to acute thermal stimulation in a behavioural test that is used to measure acute pain (Martinez et al., 2017). Together, these studies provide evidence that the NAc-PFC may be an important pathway to target for the relief of acute pain. Therefore, our findings may suggest that increased NAc-PFC coherence, a proxy measure of functional connectivity, induced by both doses of kratom possibly underlie the analgesic effects that we observed in the tail-flick test. It is important to note that while we observed changes in coherence with both doses, whether or not these underly the analgesic responses observed in the tail-flick test is unknown. Limited research has been conducted to link analgesic responses to neurophysiological changes. Nonetheless, these brain wave patterns provide a good measure for drug responses and may give insight into the addictive properties of novel compounds. Oscillations have been shown to be coupled to addictive states (Dejean et al., 2013; Zhu et al., 2019). Specifically, in a clinical study, opiate dependent patients exhibited significant reorganization of brain oscillations in all EEG oscillatory channels (Fingelkurts et al., 2006). These oscillatory adaptations are further evident in rodents upon administration of opioids (Reakkamnuan et al., 2017; Zhu et al., 2019). In particular, a recent study where rats were repeatedly administered the opioid heroin, enhanced theta band power and decreased gamma band power in the medial PFC were shown (Zhu et al., 2019).

### Oscillatory Changes Accompanying the Antidepressant-like Effects of Kratom

It was demonstrated that only the low dose of kratom had antidepressant-like properties emphasizing the important relationship between kratom dose and behavioural outcome. To our knowledge, there are no other examples of antidepressants losing effectiveness at high doses, although the effective dose of typical antidepressants is highly dependent on the individual. It is quite possible that the higher dose of kratom had additional biological effects not captured in the present study that offset the antidepressant-like properties of the drug. Ketamine, for example, while a highly effective antidepressant at low doses, induces a psychosis-like state at higher doses (Anticevic et al., 2015; Hoflich et al., 2015; Rivolta et al., 2015), and distinct dose- and time-dependent changes in neuronal oscillatory activity, particularly in the high freqency range, have been documented for this drug in both humans and animals (Rivolta et al., 2015; Manduca et al., 2020).

These findings are in line with a previous report that found that a single injection of a kratom alkaloid extract was sufficient to significantly reduce FST immobility time in mice (Kumarnsit et al., 2007b). The antidepressant-like activity of kratom was further demonstrated in a separate study conducted by Kumarnsit et al. (2007a), where intragastric administration of kratom reduced the amount of time rodents spent immobile in the tail suspension test (TST), another test commonly used to measure behavioural despair. Furthermore, a preclinical study looking solely at isolated mitragynine found that the alkaloid had dose-dependent antidepressant effects, with the higher dose (30 mg/kg) being of almost equal efficacy to that of a standard preclinical dose of fluoxetine or amitriptyline, two established antidepressants (Idayu et al., 2011). Specifically, mitragynine when administered to mice was found to significantly reduce immobility time in the FST and the TST, reductions that were comparable to mice who were administered the antidepressants (Idayu et al., 2011). These antidepressant-like effects were accompanied with a marked reduction in corticosterone concentrations signifying a role for the hypothalamic-pituitary-adrenal axis in mediating the observed effects (Idayu et al., 2011). Overall, these findings may suggest that a mechanism of action of commonly used antidepressants may be similar to that of mitragynine (Idayu et al., 2011).

Administration of high dose kratom suppressed theta power in PFC and Cg, a finding in line with another study reporting reduced theta power in cortical regions as measured by EEG (Cheaha et al., 2015). This study additionally compared those findings to that of the antidepressant fluoxetine and found the same reduction in cortical theta power (Cheaha et al., 2015). Here, we observed a reduction in theta power in response to only the high dose of kratom, and only the low dose exhibiting antidepressant properties. A reduction in Cg beta and low gamma power was also evident following repeated low dose kratom, yet the antidepressant effect was evident upon acute kratom administration, as well as following repeated dosing. Given that we were unable to demonstrate low dose-specific changes in oscillations that were present at both time points, it is possible that we simply did not capture the region-specific oscillatory changes coupled to the antidepressant effect of the drug. The hippocampus, for example, is a brain region involved in learning and memory and plays an important role in the pathophysiology of depression (Campbell and MacQueen, 2004). A recent study conducted by our group (Thériault et al., 2021) found that temporal changes in oscillations in response to chronic mild stress occurred first in the dorsal hippocampus with subsequent oscillatory changes in other brain regions that eventually culminated in the manifestation of depression-like behaviour. Other limbic brain regions implicated in the pathophysiology of depression include the amygdala and thalamus whereby functional and structural changes in these regions have been observed in depressed individuals (Pandya et al., 2012). As such, it is important to evaluate multiple different brain regions to capture relevant changes within the putative depression network.

Notably, elevated high gamma coherence between the NAc-Cg following acute and repeated injections of low dose kratom was evident. Thériault et al. (2021) similarly found an increase in NAc-Cg high gamma coherence that was evident in animals who were found to be resilient to stress and thus did not develop a depression-like phenotype following chronic daily stressors. In line with this, a low dose of ketamine administered to rats was also found to increase high gamma coherence in the NAc-Cg and these changes were postulated to be associated with a reduction of immobility time in the FST (Manduca et al., 2020). Further, alterations in the gamma frequency band have been reported to arise following pharmacological treatments that are successful in reversing symptoms of depression (Fitzgerald and Watson, 2018). Thus, it is possible that the enhancement in NAc-Cg gamma coherence observed in our study following acute and repeated low dose kratom may play a role, at least in part, in the observed antidepressant-like effects.

### Effects of Kratom on ΔFosB Expression

The repeated administration of drugs of abuse, including analgesics such as morphine, have been found to induce accumulation of ΔFosB in several brain regions (Marttila et al., 2006; Núñez et al., 2010; Li et al., 2012; Perreault et al., 2016), a process suggested to represent a molecular switch for addiction (Nestler et al., 2001). Further, it has been previously shown that overexpression of ΔFosB in the NAc of mice results in behavioural changes similar to those induced by chronic morphine administration such as rapid analgesic tolerance and enhanced drug sensitivity (Zachariou et al., 2006). Thus, these findings provide evidence that ΔFosB likely plays an important role in mediating the effects of opiates on the brain (Zachariou et al., 2006). However, although there has been controversy as to the addictive potential of kratom (Harun et al., 2015; Yusoff et al., 2016; Yusoff et al., 2017; Negus and Freeman, 2018; Hemby et al., 2019), to our knowledge there have been no studies that have evaluated kratom- or alkaloid specific-induced changes in the expression of this marker. We found no effects of the high dose of kratom on ΔFosB accumulation in any of the regions examined, a finding suggesting a lack of addictive potential for the dose and extract used. However, in the current study addictive behaviours were not explicitly evaluated, and thus such a conclusion is premature. Preclinical rodent studies have demonstrated the addiction potential of mitragynine as it was found to induce locomotor sensitization (Ismail et al., 2017) and elicit conditioned place preference thereby demonstrating that the drug has rewarding effects (Yusoff et al., 2017). However, no studies to date have evaluated the addictive properties of the extract as a whole.

In addition, in the present study overall tolerance to the effects of kratom were not evident throughout our 7-day regimen, an effect commonly seen with repeated administration of opioid analgesics, including in such tests as the tail-flick test used herein (Listos et al., 2019). The mechanism of action of opioids such as morphine is well documented in the literature, whereby morphine binds to and activates the MOR, a G protein-coupled receptor (Ream and Michael, 2011). Upon binding to the MOR, one of the induced intracellular signalling pathways results in the phosphorylation of the receptor and subsequent recruitment of the regulatory protein ß-arrestin 2 (Ream and Michael, 2011). The activation of ß-arrestin 2 has been found to contribute to morphine tolerance and mediates side effects such as respiratory depression (Caron et al., 2000; Váradi et al., 2017). Tolerance commonly arises following the repeated administration of drugs such as opioids whereby the original dose used to achieve analgesia is no longer found to be effective, therefore a larger dose must be administered to achieve the same pharmacological effects (Hurlé, 2001). Unfortunately, the exact mechanism of action of the whole kratom extract in the brain is unclear. However, the alkaloids mitragynine and 7-HMG have been identified as agonists at the MOR where they demonstrate biased activation and thus do not recruit ß-arrestin 2 (Takayama et al., 2002; Kruegel et al., 2016; Ismail et al., 2017). As such, it has been postulated that kratom may demonstrate analgesic efficacy, without bringing forth the typical life-threatening and adverse side effects of commonly prescribed opioids (Takayama et al., 2002; Kruegel et al., 2016; Ismail et al., 2017). In the present study it appears as though kratom has analgesic effects without inducing significant tolerance or ΔFosB expression, suggesting that the kratom extract may have therapeutic potential in the absence of unwanted side effects. However, further reward and addiction studies, such as those evaluating conditioned place preference or self-administration, are necessary to determine more conclusively the impact of kratom on these behaviours.

In conclusion, we showed that kratom exerted dose-dependent antidepressant-like and analgesic effects that were accompanied by frequency specific changes in neuronal oscillatory activity. In addition, the repeated administration of high dose kratom did not result in the accumulation of ΔFosB in any of the regions studied. Whereas this latter finding may indicate a lack of addictive potential, caution is warranted as only a single dose of one specific extract was evaluated. This study provides a promising direction to explore the untapped potential of kratom-based alkaloids for the management of mood and pain related disorders.

## Data Availability

The raw data supporting the conclusions of this article will be made available by the authors, without undue reservation.
