# Supplementary material for: The Antidepressant-Like and Analgesic Effects of Kratom Alkaloids are accompanied by Changes in Low Frequency Oscillations but not ΔFosB Accumulation
Source: Front Pharmacol. 2021 Aug 3;12:696461. doi: 10.3389/fphar.2021.696461 (PMC8369573; doi:10.3389/fphar.2021.696461)
Supplement: Supplementary file 1 [file DataSheet1.PDF]

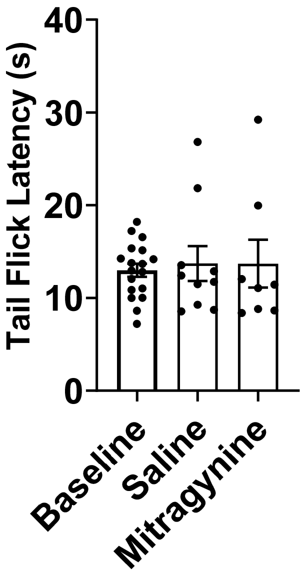

Figure S1. Effect of mitragynine on tail flick latency. Mitragynine (10 mg/kg, i.p.) had no effect on tail flick latency.

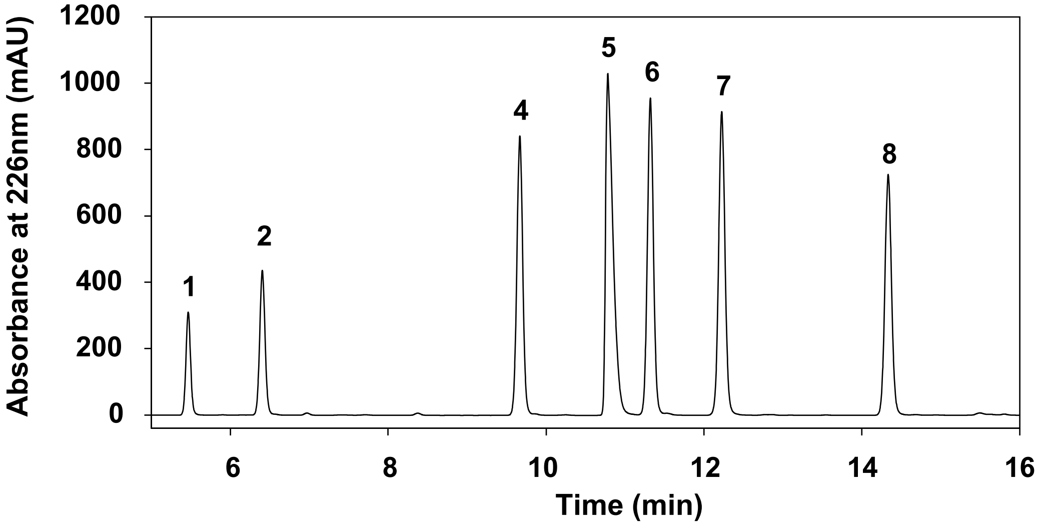

Figure S2. HPLC chromatogram of alkaloid authentic standards containing seven reference substances at 226 nm. The highlighted peaks represent respectively: 1, mitraphylline; 2, 7-hydroxymitragynine; 4, ajmalicine; 5, speciociliatine; 6, paynantheine; 7, speciogynine, 8, mitragynine.

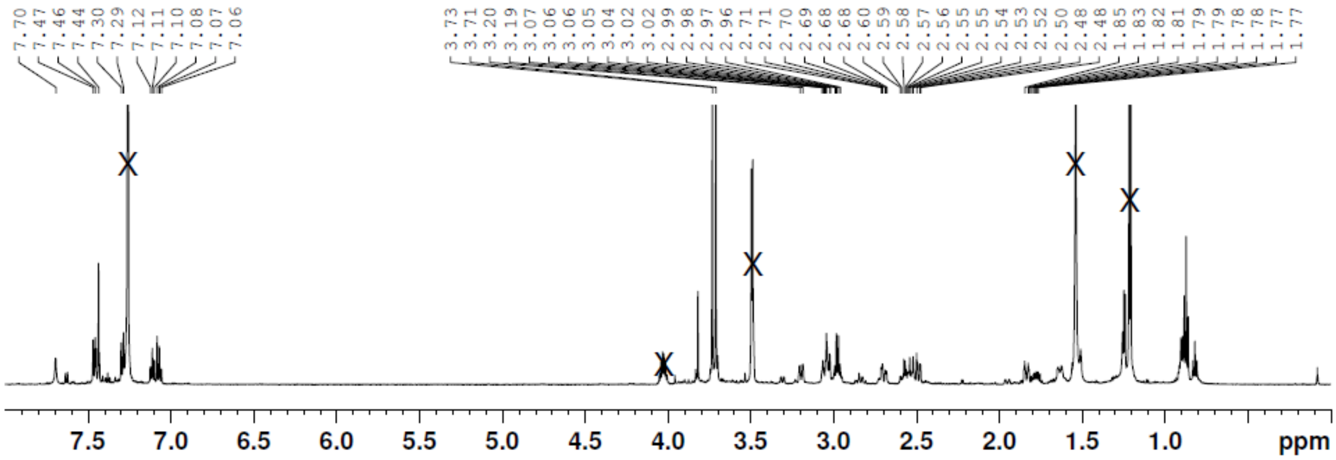

Figure S3. ^{1}H NMR spectrum for fraction 9 ($CDCl_3$, 600 MHz). Two alkaloids are present in a 4:1 concentration ratio (as determined by integration of the aromatic peaks). Chemical shifts for the major alkaloid are in excellent agreement with those reported for corynantheidine (Lounasmaa et al., Mizuno et al.). The minor alkaloid was not identified. The residual solvent peak at 7.26 ppm, trace methanol peak at 3.49 ppm, trace water peak at 1.55 ppm, and trace isopropanol peaks at 4.02 and 1.21 ppm have been marked with an X.

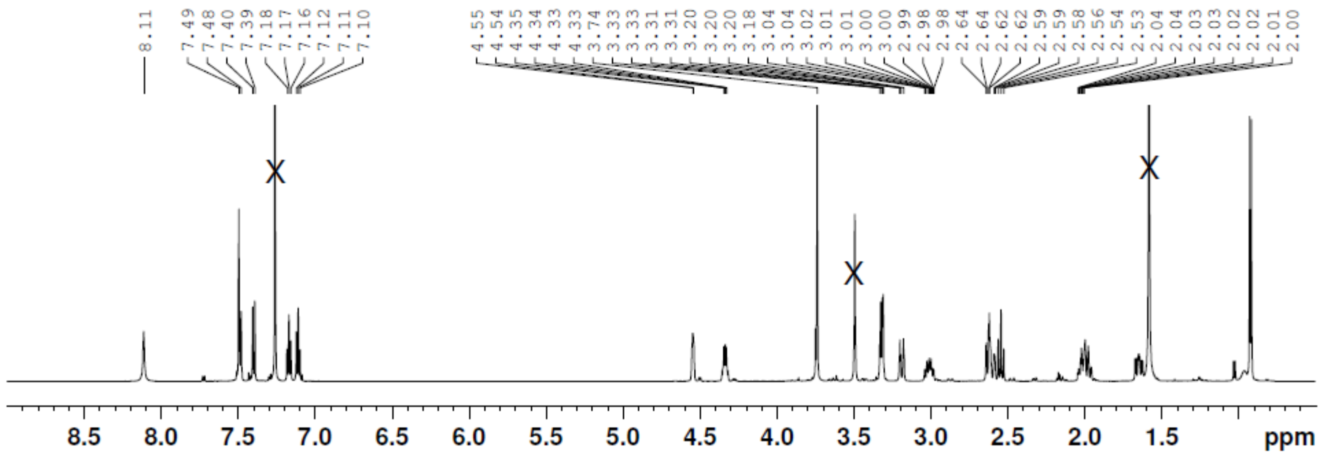

Figure S4. ^{1}H NMR spectrum for fraction 3 ($CDCl_3$, 600 MHz). The spectrum is in excellent agreement with that reported for 3-isoajmalicine (Philipson et al., Carbonezi et al.). The residual solvent peak at 7.26 ppm, trace methanol peak at 3.49 ppm, and trace water peak at 1.55 ppm have been marked with an X.
